# Outcomes of a controlled trial with visiting therapy dog teams on pain in adults in an emergency department

**DOI:** 10.1371/journal.pone.0262599

**Published:** 2022-03-09

**Authors:** Ben Carey, Colleen Anne Dell, James Stempien, Susan Tupper, Betty Rohr, Eloise Carr, Maria Cruz, Sharon Acoose, Peter Butt, Lindsey Broberg, Lisa Collard, Logan Fele-Slaferek, Cathie Fornssler, Donna Goodridge, Janet Gunderson, Holly McKenzie, Joe Rubin, Jason Shand, Jane Smith, Jason Trask, Kerry Ukrainetz, Simona Meier

**Affiliations:** 1 Department of Sociology, University of Saskatchewan, Saskatoon, Canada; 2 College of Medicine, University of Saskatchewan, Saskatoon, Canada; 3 Quality, Safety & Standards, Saskatchewan Health Authority, Saskatoon, Saskatchewan, Canada; 4 Faculty of Nursing University of Calgary, Calgary, Alberta, Canada; 5 School of Indigenous Social Work, First Nations University of Canada, Saskatoon, Saskatchewan, Canada; 6 Emergency Services, Saskatchewan Health Authority, Saskatoon, Saskatchewan, Canada; 7 College of Arts & Science, University of Saskatchewan, Saskatoon, Canada; 8 Saskatchewan Centre for Patient Oriented Research, University of Saskatchewan, Saskatoon, Saskatchewan, Canada; 9 College of Nursing & Respirology, Critical Care and Sleep Medicine, University of Saskatchewan, Saskatoon, Saskatchewan, Canada; 10 Veterinary Microbiology, University of Saskatchewan, Saskatoon, Saskatchewan, Canada; 11 Clinical Analyst, Saskatchewan Health Authority, Saskatoon, Saskatchewan, Canada; 12 St. John Ambulance, Saskatoon, Saskatchewan, Canada; 13 Clinical Research Professional Clinical Trial Support Unit, University of Saskatchewan, Saskatoon, Canada; Memorial University of Newfoundland, CANADA

## Abstract

**Context:**

Pain is a primary reason individuals attend an Emergency Department (ED), and its management is a concern.

**Objectives:**

Change in symptoms and physiologic variables at 3 time points pre-post a ten-minute St. John Ambulance therapy dog team visit compared to no visit in ED patients who experienced pain.

**Design, setting and participants:**

Using a controlled clinical trial design, pain, anxiety, depression and well-being were measured with the Edmonton Symptom Assessment System (revised version) (ESAS-r) 11-point rating scales before, immediately after, and 20 minutes post- therapy dog team visit with Royal University Hospital ED patients participating in the study (n = 97). Blood pressure and heart rate were recorded at the time points. Control data was gathered twice (30 minutes apart) for comparison (n = 101). There were no group differences in age, gender or ethnicity among the control and intervention groups (respectively mean age 59.5/57.2, ethnicity 77.2% Caucasian/87.6%, female 43.6% /39.2%, male 56.4%/60.8%,).

**Intervention:**

10 minute therapy dog team visit in addition to usual care.

**Main outcome measures:**

Change in reported pain from pre and post therapy dog team visit and comparison with a control group.

**Results:**

A two-way ANOVA was conducted to compare group effects. Significant pre- post-intervention differences were noted in pain for the intervention (mean change^int.^ = -0.9, SD = 2.05, p = .004, 95% confidence interval [CI] = [0.42, 1.32], η_p_^2^ = 04) but not the control group. Anxiety (mean change^int.^ = -1.13, SD = 2.80, p = .005, 95% CI = [0.56, 1.64], η_p_^2^ = .04), depression (mean change^int.^ = -0.72, SD = 1.71, p = .002, 95% CI = [0.39, 1.11], ηp^2^ = .047), and well-being ratings (mean change^int.^ = -0.87, SD = 1.84, p < .001, 95% CI = [0.49, 1.25], ηp^2^ = .07) similarly improved for the intervention group only. There were no pre-post intervention differences in blood pressure or heart rate for either group. Strong responders to the intervention (i.e. >50% reduction) were observed for pain (43%), anxiety (48%), depression (46%), and well-being (41%).

**Conclusions:**

Clinically significant changes in pain as well as significant changes in anxiety, depression and well-being were observed in the therapy dog intervention compared to control. The findings of this novel study contribute important knowledge towards the potential value of ED therapy dogs to affect patients’ experience of pain, and related measures of anxiety, depression and well-being.

**Trial registration:**

This controlled clinical trial is registered with ClinicalTrials.gov, registration number NCT04727749.

## Introduction

Pain is both an emotional and a sensory experience that is unpleasant and specific to an individual [1, 2]. The primary reason patients visit a hospital Emergency Department (ED) is to address pain and these account for approximately 80% of all visits [3, 4]. An ongoing concern with quality of care in Eds is that patient pain is inadequately managed, in part because of long wait times [5]. It is also recognized that experiencing anxiety in the ED can negatively impact patients’ pain and the perception of wait times [6–8]. The aim of this study is to determine the effect that visiting therapy dog teams have on hospital ED patient pain following a controlled clinical trial format.

The ED setting has also been linked as a contributor to patient pain. Common environmental stressors, such as constant bright lighting and noise levels [9, 10], may disrupt ED patients’ rest patterns. This can slow down the recovery process and prolong patients’ symptoms of pain [11, 12]. Moreover, as waiting for extended periods of time is typical of the ED patient experience, patients’ sensory awareness may be heightened and they may lack sufficient distraction, both which may amplify their perceptions of pain [9]. Therapy dog and handler teams visit patients in public health settings, including hospitals, for motivational, educational, therapeutic and recreational benefit [13]. These visits have been documented to benefit the psychological health of individuals by reducing stress, anxiety, depression and feelings of loneliness [14–22]. A study by Harper et al. [23] found that involving visiting therapy dogs in client care plans immediately following joint replacement surgery improved participants’ pain scores.

The first therapy dog team to visit an ED in Canada was at the Royal University Hospital (RUH) in Saskatoon, Saskatchewan in 2016. These visits started in part to offer distraction to patients from long wait times [24]. Since then, at least eight additional Eds have welcomed visiting therapy dog teams in Canada; i.e., St. Paul’s Hospital, City Hospital, Jim Pattison Children’s Hospital (Saskatoon, SK), Queen Elizabeth Hospital (Charlottetown, PEI), South Health Campus (Calgary, AB), Queensway Carleton Hospital (Ottawa, ON), Michael Garron Hospital (Toronto, ON) and Thunder Bay Regional Health Sciences Center (Thunder Bay, ON).

To date, few studies have been undertaken on visiting therapy dog teams in Eds. A 2012 study by Nahm et al. [25] found that both patients and staff were supportive of visiting therapy dogs in a Midwest hospital in the USA. A recent clinical trial, also in the USA, of visiting therapy dogs in an ED found the visits assisted with reducing patient anxiety [26]. A case study by Dell et al. [24] of patients in a Canadian ED revealed that visiting with a therapy dog improved patients’ perceived levels of comfort and distress and it was a welcome distraction for patients from a stressful ED environment. These same authors surveyed patients waiting in an ED about whether they would want to be visited by a therapy dog, and the vast majority agreed and were of the opinion that patients may want to visit with a therapy dog “to reduce anxiety (92%) and frustration (87%) as well as to increase comfort (90%) and satisfaction (90%) and to a lesser extent to reduce pain (59%)” [27].

Research in the visiting therapy dog field exists and is growing but remains commonly criticized for a lack of control groupings, small sample sizes and absence of quantitative data collection [18, 28–31]. The current study is designed to address these criticisms in part and add to the knowledge base and methodological rigor of the field with the contribution of a controlled trial. It focuses on understanding the impact of an innovative psychosocial health intervention–a visiting therapy dog team–on patients’ experiences of pain and related psychosocial and physiological variables in the ED. This study also contributes a gendered analysis to the field of study. Further, no research to date has isolated the presence of the therapy dog from the handler in a visit, and although we did not either, we do acknowledge they are a team and attempted to standardize the handler’s role in the visit to the best of our ability (see below for a typical visit).

## Methods

A controlled clinical trial was conducted with a single intervention and control group and pre-post data collection. Patients were allocated to either the experimental or control group according to day of the week, in order to prevent exchanges of therapy dog teams with control groups. Days were randomly assigned as either control or dog team prior to patient enrolment to rule out any selection bias and other possible known or unknown confounding variables. Every eligible patient in the ED was asked to participate if able. During days of the control group, only the researchers were not blind to the control assignment.

The intervention group received a 10-minute visit with a St. John Ambulance certified therapy dog and handler in addition to usual care. More details on the teams and visit are provided below. A research assistant collected data from participants randomized to the intervention group immediately before the 10 minute visit, immediately after and 20 minutes post-visit. For the control group, data were gathered twice, with a 30 minute interval between.

### Patient oriented research

Our study adopted a patient oriented approach (POR), recognizing the importance of accounting for patient experiences in healthcare research [32, 33]. Our study team is comprised of clinicians, researchers, therapy dog handlers and patient advisors. The patient advisors guided the content and data collection tools for the ED setting, and participated in data interpretation and dissemination of findings.

### Site selection

The Royal University Hospital Emergency Department (RUH ED) was chosen as the study location because of its longstanding visiting therapy dog program. There are established partnerships between RUH administration, the St. John Ambulance Therapy Dog program, patient advisors and researchers, and Saskatchewan Health Authority (Saskatoon) Infection Prevention & Control to undertake a complex and time-intensive study in the ED. RUH is a major teaching hospital at the University of Saskatchewan, an urban center, and is the trauma and tertiary care center for the province. RUH is the busiest ED in the province, averaging 150–200 adult visits per 24 hours.

### Participant selection

In order to ensure successful blinding of participants, randomization was achieved by randomizing days that the therapy dog teams would visit the ED. All participants recruited on a given intervention or control day would be allocated to that group. A Research Assistant approached potential participants asking if they would like to participate in a pain study, unless they were sleeping or under constant treatment. Eligibility criteria included: over the age of 18, able to provide consent, presented to the ED with some form of discomfort, and a Canadian Triage and Acuity Score (CTAS) of 2–5 (a score of 1 is resuscitation). Participants in the intervention group also had to be willing to visit with a therapy dog team. Control group participants were informed about the purpose of the study after data collection was complete. All participants were located in individual curtained-off beds or rooms in the ED. The patients were waiting to be seen by a physician, had treatment in progress, or were admitted to the hospital and waiting for a unit bed. All patients who fit the participant selection criteria and could be approached without interfering with their care were recruited. No activities were used to increase recruitment or compliance in this study.

### Therapy dog teams

A therapy dog team consisted of a St. John Ambulance certified (tested and passed) handler and dog, which deemed them suitable for public visiting. In addition to regular St. John Ambulance therapy dog program visiting policies and procedures (e.g., hygienic dog grooming), supplementary guidelines were developed to ensure the health and welfare of patients, staff, and the therapy dog and handler entering the ED. These included pre- and post-visit hand sanitation, protective padding for bedding surfaces the dogs interact on, St. John Ambulance and Saskatchewan Health Authority (Saskatoon) volunteer training and paperwork, shadowing a therapy dog team in the ED prior to commencing solo visits, and placing a standing poster by the ED entrance when a therapy dog is present to inform individuals who do not wish to come in contact with a dog.

### Procedures

Recruitment and follow-up took place between June 7 to September 20, 2019 from a convenience sample in the RUH ED five days a week during 90-minute windows at both peak and low patient ED wait times. Upon receiving consent for participating, baseline measures were taken. On average, a therapy dog team visit with the intervention group was ten minutes in duration, which is a typical therapy dog team visit length in a healthcare setting [34, 35].

A standardized visiting protocol across patients was developed and followed closely for this study. For the intervention group, patient greets the therapy dog, handler shares information about the therapy dog, asks about patient’s pets, and offers a trading card of the therapy dog at the conclusion of the visit. The majority of the visits involve the patient taking the lead in the conversation with the handler actively listening. The control group was blinded by completing a consent form that did not specify the therapy dog focus of the study and they were asked to complete a second consent form after data collection which explained the study in more detail, including that a comparison group visited with a therapy dog team.

### Data collection

Pain severity, anxiety, depression, and general well-being were measured with 11-point numeric rating scales using items from the Edmonton Symptom Assessment System (revised version) (ESAS-r) in which higher ratings indicated worse outcomes [36, 37]. The ESAS originated in 1991 and is a psychometrically validated symptom assessment instrument that has low responder burden [38]. The ESAS has since been updated and studies have been undertaken to establish its psychometric validity [38]. The ESAS-r is recommended for use by the Initiative on Methods, Measurement, and Pain Assessment in Clinical Trials [39]. Location of pain was reported using a body diagram. The standard wording for the questions were: “Can you tell me all of the areas on your body where you are feeling pain or discomfort. Can you rate your pain on a scale of 0 (indicating no pain) to 10 (indicating the worst possible pain)”, “Can you describe your Anxiety, or feeling of nervousness on a scale from 0 being no anxiety, or feeling of nervousness to 10 being worst possible anxiety or feeling of nervousness?”, “Can you describe your Depression, or feeling of sadness on a scale from 0 being no Depression, or feeling of sadness to 10 being worst possible Depression, or feeling of sadness?”, and “Can you describe your Well-being, how you feel overall on a scale from 0 being best Well-being, or feeling the best possible overall to 10 being worst possible Well-being, or feeling the worst possible overall?”. Physiological measures of heart rate and mean arterial blood pressure were measured using roaming vital sign monitors or bedside cardiorespiratory monitors. There was no change in a participant’s device during their trial. Data collection did not exceed 10 minutes per patient (see Table 1). Testing was done on both days by three Research Assistants (Ras) from a pool of 10, RA# 1 and #2 were graduate or undergraduate multidisciplinary students at the University of Saskatchewan and RA# 3 was a medical or nursing student at the University of Saskatchewan College of Medicine or College of Nursing respectively. There were no deviations from the original protocol other than deciding not to collect data on the Human-Animal Interaction Scale or patient perception of treatment by healthcare workers due to time constraints (i.e., did not want data collection to be longer than the visit itself for the intervention group) [40].

**Table 1:** Data collection steps for intervention & control groups.

Intervention Group	
Step 1	Research Assistant (RA)#1 –Do you want to visit with a dog? Are you here for pain?
	RA#3 –Check Medicine Administration Record (MAR) for whether took pain medication in the last hour and document.
Step 2a	RA#2 –Ask pain, anxiety, depression, well-being and demographic questions.
Step 2b	
	RA#3 –Collect Heart Rate (HR) and Blood Pressure (BP)
	Intervention in addition to usual care.
	Post-intervention (at conclusion of the intervention).
Step 3a	RA#3 –Collect HR and BP
Step 3b	RA#2 –Ask pain, anxiety, depression and well-being questions.
**Control Group**	
Step 1	RA#1 –Do you want to participate in a study? Are you here for pain?
	RA#3 –Check MAR for whether took pain medication in the last hour and document.
Step 2a	RA#2 –Ask pain, anxiety, depression, well-being and demographic questions.
Step 2b	
	RA#3 –Collect HR and BP.
	No Intervention–Care as Usual.
Step 3	Post-intervention (10 minutes later /conclusion of the non-intervention).
Step 4a	RA#3 –Collect HR and BP.
Step 4b	RA#2 –Ask pain and anxiety, depression and well-being questions.

### Sample size

The minimum sample size to detect significant pain score difference between the visiting therapy dog team and control group, with a level of significance = 0.05 and power = 80%, was determined to be 100 participants. The Clinical Research Support Unit in the College of Medicine at the University of Saskatchewan assisted with calculation of the sample size. To calculate this, findings from a pilot study were drawn upon [24], assuming the mean pain score would be 4.5 in the intervention group (after therapy dog team visit) and 5.5 in the control group (at second measurement), respectively.

A total of 101 participants were randomly recruited into the control group and 97 into the intervention group; 3 control group participants were excluded from the final analysis because they were unable to complete the measurements because of hospital care being their priority and 11 intervention group participants were excluded (10 unable to complete the measurements and 1 declined to participate after the initial data collection). In total, 355 ED patients were approached by a research assistant to take part in the study.

**Fig 1:**
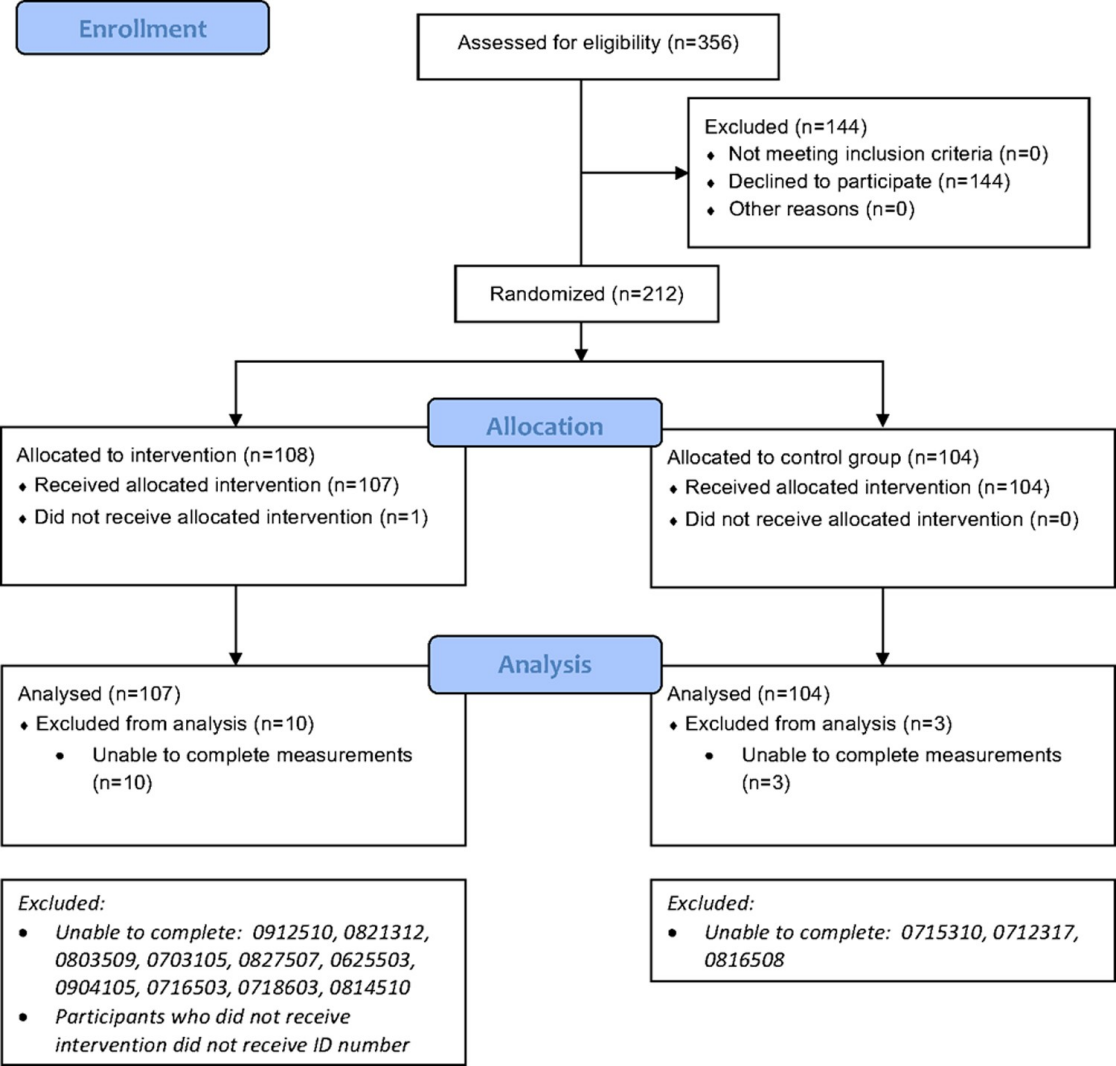
CONSORT 2010 flow diagram.

When our team initially designed this study, the minimum sample size was determined to be 122, and this was recorded in our ethics application. However, with updated data from our pilot study, and drawing on the expertise of the Clinical Research Support Unit, the sample size was recalculated, as described above, and determined to be 100 to detect statistically significant treatment effects. Consequently, our research ethics application incorrectly states the original target sample of 122. Our clinicaltrial.gov registry (NCT04727749) accurately identified 198 patients for total recruitment for the visiting therapy dog team and control groups of patients [40]. Fig 1 presents the CONSORT diagram.

### Statistical analysis

Quantitative data analysis was conducted using IBM SPSS Version 25.0, to assess the overall effect of the therapy dog team intervention using a two-way independent ANOVA test to compare mean difference in change scores (i.e., post-treatment measure minus pre-treatment measure produced a change score for each participant) for each dependent variable across gender. The dependent variables were pain, anxiety, depression, and well-being. Prior to applying the ANOVA, we used Levene’s test of equality to examine for homogeneity of variance.

Responder analyses, similar to IMMPACT (Initiative on Methods, Measurement, and Pain Assessment in Clinical Trials) [41], were conducted to further explore the proportion of participants experiencing a minimal or moderate degree of change in pain. Although guidelines for responder analyses have not been recommended for psychosocial variables, exploratory analysis using the same thresholds for response were applied to anxiety, depression, and well-being scores [39]. Medcalc, an online tool was used for determining differences between the two proportions. The following thresholds for response were used: Less than a 30% reduction in pain, anxiety, depression or well-being was considered a minimal response. A between 30% and 49% reduction in pain was considered a moderate response. A response of 50% or greater reduction in pain was considered strong. For example, if a participant’s baseline pain score was 6/10, a 4/10 pain score in the follow-up measurement would be considered a moderate response (change of 2/6 = 33.3%), and a 3/10 would be considered a strong response (change of 3/6 = 50%) [42]. No change or increase in pain were also examined. Two sample proportion chi square tests, as recommended by Campbell [43] and Richardson [44], were conducted using Medcalc’s comparison of proportions calculator (medcal.org) to determine if there was a difference in proportion of responders between the therapy dog team group and the control group for each category. Note that cases were excluded from response analysis where Time 1 was 0/10 pain, anxiety, depression, or well-being to delineate from no change in response when there was some experience of the variable to begin with.

Finally, an examination of the potential influence of pain medication was conducted. The research assistants, who were medical residents, reviewed the patients’ charts and recorded all pain medications. An examination of the potential influence of pain medication was conducted. All pain medications, regardless of strength, were considered. Medications were evaluated for timing of both onset and peak of medication effectiveness based on typical pharmacokinetics. Pain medications were examined as a potential confounding variable if the timing of onset or peak of medication effectiveness occurred between the baseline and post-measurements. There was only one case with a potential for medication influencing pain response; therefore, no adjustments were made to the analysis.

### Ethics

Operational approval for this research was secured from the Saskatchewan Health Region (Saskatoon) and ethics approval was granted from the University of Saskatchewan Human Behavioural (#1253) and Animal (#20130115) Research Ethics Boards. All participants provided informed written, or when not possible because of a physical ailment, verbal consent prior to taking part in the study. This study was not registered with clinicaltrials.gov prior to data collection as the research team was unaware that it fit the conditions to be considered a clinical trial. The authors confirm that it has since been registered and all ongoing and related trials for this intervention are registered (NCT04727749). No important harms or unintended effects were reported.

## Results

### Participants

Among the 211 participating patients, 198 were available for post data collection (control group n = 101, 51%). The reasons for the 144 ED patients who were approached but chose not to participate (control group n = 59, 41%) included: 53 (36.8%) not interested, 46 (32%) do not feel up to it, 5 (3.5%) allergic, 3 (2.1%) do not like dogs, 2 (1.4%) fear of dogs, 8 (5.6%) declined to answer, and 27 (18.8%) other reasons, such as busy/had a visitor, scheduled for a test in the near future, and about to be discharged Dog specific responses are specific to the intervention group. There is no reason to consider that data collected at baseline for the study population would be different than that for the target population of interest attending the RUH ED for pain.

There were no group differences in age, gender or ethnicity among the intervention and control participant groups (see Table 2). The control group mean age was 59.5 years compared to 57.2 years in the therapy dog intervention group; there was no statistically significant difference of mean age by group as determined by a t-test (*t(*1,195) = 0.787, *p* = 0.44). Age was not reported by one participant in the intervention group. The proportion of females to males was approximately 40% to 60% and was determined to not be significantly different between the groups by a z-test of two proportions (*z* = 0.63, *p* = 0.53). No gender non-binary identities were reported. A large majority (82%) of participants identified their ethnic origin as Caucasian, (control at 77% and therapy dog intervention at 88%) and was determined to not be significantly different by a z-test of two proportions (z = 1.92, *p* = 0.056).

**Table 2:** Demographics by group.

		Control	Dog Intervention	Total group
Age	Mean Age	59.5	57.2	58.4
Gender	Female n (%)	44^a^ (43.6%)	38^a^ (39.2%)	82 (41.4%)
	Male n (%)	57^a^ (56.4%)	59^a^ (60.8%)	116 (58.6%)
Ethnicity	Indigenous n (%)	18 (17.8%)	9 (9.3%)	27 (13.6%)
	Caucasian n (%)	78 (77.2%)	85 (87.6%)	163 (82.3%)
	Other n (%)	5 (5.0%)	3 (3.1%)	8 (4.0%)
	Total n (%)	101 (100.0%)	97 (100.0%)	198 (100.0%)

^a^ z-test comparing column proportions did not differ significantly at 0.05 level.

The majority (69.2%) of participants were admitted and awaiting a hospital bed, with no difference between groups (proportion z-test at 0.05 level) (see Table 3).

**Table 3:** Admitted/waiting hospital bed by group.

	Control	Dog Intervention	Total
Admitted/Waiting Hospital Bed (n) (%)	69^a^ (68.3%)	68^a^ (70.1%)	137 (69.2%)
Other (n) (%)	32^a^ (31.7%)	29^a^ (29.9%)	61 (30.8%)
Total (n) (%)	101 (100.0%)	97 (100.0%)	198 (100.0%)

^a^ z-test comparing column proportions did not differ significantly at 0.05.

The majority (65, 67%) of the therapy dog intervention participants self-reported a lot of experience/history with owning dogs (e.g., own or owned a dog); 28 (29%) had some (typically indicating they had a dog in the past) and 4 (4%) had none. At the time of data collection, 59 (61%) did not live with a companion dog. 33 (34%) of the dog intervention group self-reported to have had a lot of experience with having companion animals other than dogs, 40 (41%) some and 22 (23%) none. Forty-one percent of participants had a family member or other non-hospital support with them during data collection, and with no difference between groups.

### Pain

All participants reported pain at the time of recruitment into the study (yes/no response option). After recruitment, when participants were asked to score their pain on a 0–10 scale for the baseline measure, a minority of participants (26.3%) provided a score of 0, indicating no pain RA#1 asked participants in the study if they are experiencing pain or discomfort. RA#2 asked patients about their levels of pain. Some patients reported pain to the first RA and then 0 as a level of pain to the second RA. Some patients also described their experience as emotional pain to RA#1 but then rated it as 0 in response to the ESAS-R “tell me what part of your body is in pain” question, yet still reported their anxiety and depression as 10. A slightly higher proportion of the dog intervention group (80.4%) reported pain (a non-zero score on the ESAS pain item) compared to the control group (67.3%). There was no significant main effect of gender on the between group differences (See Table 4).

**Table 4:** Descriptive statistics for pain.

Factor	Category	Change Score	SD	N
Mean				
Gender	Male	-0.56	2.20	116
	Female	-0.22	2.26	81
Group	Control	-0.03	2.30	101
	Dog Intervention	0.90	2.05	96

In contrast, there was a statistically significant main (albeit small) effect of the therapy dog intervention on participants’ pain ratings. Participants in the therapy dog team group rated pain significantly lower than those in the control group at the post-intervention measurement. In addition, no significant interaction was found between gender and the intervention. These findings suggest that the therapy dog intervention had a positive effect on reducing participant pain, and that this effect is similar for people of both male and female genders (See Table 5).

**Table 5:** ANOVA summary table for pain.

Source	*df*	MS	*F*	*p*	Effect Size
A Gender	1	3.89	0.81	.369	0.004
B Group	1	41.04	8.54	.004	0.042
A x B Interaction	1	0.51	0.11	.744	0.001
Within groups	193				
Total	196				

*Note*. MS  =  Mean squares, effect size  =  partial *η2*.

#### Pain response

There was no statistically significant difference in proportions of participants in each pain response category between groups (see Table 6). There is a trend in that more cases in the therapy dog team group had a strong response and more cases in the control group had no change (see Table 6). Participants with no pain, a recorded pain rating of 0 at pre and post, were excluded from pain response analysis (42 control, 37 intervention).

**Table 6:** Proportion of participants in each pain change response category and chi-square statistics.

Pain	No change in pain	Low 1 to 29%	Mod. 30–49%	Strong 50%+	Increase	Total
Control *n (%)*	25 (42.37)	4 (6.78)	1 (1.69)	18 (30.51)	11 (18.64)	59
Dog Inter *n (%)*	17 (28.33)	6 (10.00)	4 (6.67)	26 (43.33)	7 (11.67)	60
*X*^*2*^	2.55	0.40	1.80	2.10	1.12	
*p-*value	*0*.*111 *	*0*.*529 *	*0*.*178 *	*0*.*150 *	*0*.*291 *	
CI 95%	-3.0% to 30.1%	-7.6% to 14.2%	-3.4% to 14.3%	-4.4% to 29.0%	-6.2% to 20.1%	

#### Pain response and pain medication

The majority (77%) of participants did not receive pain medication. Only 1 participant may have experienced influence of pain medication (onset of medication at baseline and peaking at follow-up). No adjustment was made to the analysis.

### Anxiety

There was a significant main (albeit small) effect of gender influence on anxiety ratings. Males overall rated a greater reduction in anxiety compared to females. Anxiety scores for females in the control group increased at follow-up (mean change in anxiety^control^ = -0.64, SD = 2.75) compared to a mean reduction in anxiety in the therapy dog team group (mean change in anxiety^int.^ = 0.95, SD = 2.39). (See Tables 7 and 8).

**Table 7:** Descriptive statistics for anxiety.

Factor	Category	Mean	SD	N
Gender	Male	0.97	2.63	116
	Female	0.09	2.70	81
Group	Control	0.11	2.60	101
	Dog Intervention	1.13	2.46	96

**Table 8:** ANOVA summary table for anxiety.

Source	*df*	MS	*F*	*p*	Effect Size
A Gender	1	31.05	4.63	.033	0.023
B Group	1	54.11	7.95	.005	0.040
A x B Interaction	1	12.58	1.85	.176	0.009
Within groups	193				
Total	196				

*Note*.—MS  =  Mean squares, effect size  =  partial *η2*.

There was a significant main (albeit small) effect of the therapy dog intervention on participants’ anxiety ratings (See Table 9). Participants in the therapy dog team group rated anxiety significantly lower at post-intervention than those in the control group. In addition, no significant interaction was found between gender and the intervention (See Table 8).

**Table 9:** Proportion of participants in each pain change response category and chi-square statistics.

Anxiety	No change	1 to 29%	30–49%	50%+	Increase	Total
Control %	41.67%	10.00%	10.00%	26.67%	11.67%	60
Dog Inter %	14.52%	19.35%	11.29%	48.39%	6.45%	62
*X*^*2*^	11.09	2.10	0.05	6.07	1.00	
*p-*value	* **0.001**	0.147	0.818	* **0.014**	0.316	
CI 95%	11.3% to 41.4%	-3.5% to 22.0%	-10.4% to 12.8%	4.5% to 37.2%	-5.5% to 16.4%	

* significant at alpha = 0.05.

#### Anxiety response

The control group had a statistically significant larger proportion of participants who had no reduction in anxiety at follow-up compared to the therapy dog team group. Participants with no anxiety, i.e., a recorded anxiety rating of 0 at pre and post, were excluded from anxiety response analysis (41 control, 35 intervention). (see Table 9).

### Depression

There was no significant main effect of gender on depression change scores, with males and females indicating similar change (See Table 10). Note that 104 of the study participants (58 from Control group and 46 from Dog Intervention Group) had a rating of zero depression at pre-measurement and 112 at post-measurement. Also, there was an assumption violation of homogeneity of variance as Levene’s test was significant [F(3,193) = 3.70, p< .05]. No adjustments were made to the analysis, but it is noted that the male data and the control data dispersions had a greater frequency of the data at no change. In addition, half (53%) of the participants did not have depression at pre-measurement.

**Table 10:** Descriptive statistics for depression.

Factor	Category	Mean	SD	N
Gender	Male	0.36	1.34	116
	Female	0.31	2.27	81
Group	Control	-0.02	1.77	101
	Dog Intervention	0.72	1.71	96

There was a significant main (albeit small) effect of the therapy dog intervention on participants’ depression ratings. Participants in the therapy dog team group rated depression significantly lower at post intervention than those in the control group (See Table 10). In addition, no significant interaction was found between gender and the intervention (See Table 11).

**Table 11:** ANOVA summary table for depression.

Source	*df*	MS	*F*	*p*	Effect Size
A Gender	1	< .001	< .001	.99	< .001
B Group	1	29.05	9.53	.002	0.047
A x B Interaction	1	2.23	0.73	.393	0.004
Within groups	193				
Total	196				

*Note*.—MS  =  Mean squares, effect size  =  partial *η2*.

#### Depression response

There was a statistically significant difference in the proportion of depression response in the no change category to support that there was a larger proportion of the control group that had no response in depression reduction at Time 2. A larger proportion in the therapy dog team group had indicated a stronger response to reduction in depression following the therapy dog intervention. Participants with no depression, a recorded depression rating of 0 at pre and post, were excluded from depression response analysis (see Table 12). (58 control, 47 intervention).

**Table 12:** Proportion of participants in each change response category and chi-square statistics.

Depression	No change	1 to 29%	30–49%	50%+	Increase	Total
Control %	46.51%	4.65%	4.65%	25.58%	18.60%	43
Dog Inter %	24.00%	16.00%	8.00%	46.00%	6.00%	50
*X*^*2*^	5.14	3.07	0.43	4.11	3.48	
*p-*value	* **0.024**	0.797	0.514	* **0.043**	0.062	
CI 95%	3.1% to 40.0%	-1.9% to 24.3%	-8.5% to 14.7%	0.8% to 37.7%	-0.9% to 27.2%	

* significant at alpha = 0.05.

### Well-being

There was no significant main effect of gender, with males and females indicating similar change in well-being scores. There was a significant main effect of the therapy dog intervention on participants’ well-being ratings (medium effect size; partial *η2 = 0*.*072*). Participants in the therapy dog team group rated well-being significantly better post intervention than those in the control group (See Table 13). In addition, no significant interaction was found between gender and the intervention (see Table 14).

**Table 13:** Descriptive statistics for wellbeing.

Factor	Category	Mean	SD	N
Gender	Male	0.47	1.80	116
	Female	0.19	2.04	81
Group	Control	-0.14	1.84	101
	Dog Intervention	0.87	1.84	96

**Table 14:** ANOVA summary table for wellbeing.

Source	*df*	MS	*F*	*p*	Effect Size
A Gender	1	2.32	0.68	.410	0.004
B Group	1	52.02	15.00	< .001	0.072
A x B Interaction	1	2.67	0.79	.376	0.004
Within groups	193				
Total	196				

*Note*.—MS  =  Mean squares, effect size  =  partial *η2*.

#### Well-being response

There was a statistically significant larger proportion of the control group that had no change in their response to well-being at Time 2. Conversely, a larger proportion in the therapy dog team group had indicated a stronger response to increased well-being following the therapy dog intervention (see Table 15).

**Table 15:** Proportion of participants in each change response category and chi-square statistics.

Well-being	No change	1 to 29%	30–49%	50%+	Decrease	Total
Control %	54.46%	0.99%	2.97%	19.80%	21.78%	101
Dog Inter %	37.89%	6.32%	3.16%	41.05%	11.58%	95
*X*^*2*^	5.38	4.02	0.01	10.45	3.62	
*p-*value	* **0.020**	* **0.045 **	0.920	* **0.003**	0.057	
CI 95%	2.6% to 29.6%	-0.2% to 12.2%	-5.6% to 6.3%	8.8% to 33.3%	-0.4% to 20.5%	

* significant at alpha = 0.05.

### Blood pressure

There was no significant main effect of gender, with males and females indicating similar change in mean arterial blood pressure scores (See Table 17). Also, there was an assumption violation of homogeneity of variance as Levene’s test was significant. [F(3,194) = 3.36, p< .05]. No adjustments were made to the analysis.

There was no significant main effect of the therapy dog condition, indicating similar change in MAP (mean arterial blood pressure) scores. In addition, no significant interaction was found between gender and the intervention (see Tables 16 & 17).

**Table 16:** Descriptive statistics for blood pressure.

Factor	Category	Mean	SD	N
Gender	Male	0.84	7.04	116
	Female	-0.05	12.08	81
Group	Control	-0.04	8.07	101
	Dog Intervention	1.01	10.70	97

**Table 17:** ANOVA summary table for blood pressure.

Source	*df*	MS	*F*	*p*	Effect Size
A Gender	1	32.39	0.36	0.55	.002
B Group	1	62.36	0.69	.41	.004
A x B Interaction	1	23.39	0.26	.61	0.001
Within groups	194				
Total	197				

*Note*.—MS  =  Mean squares, effect size  =  partial *η2*.

### Heart rate

There was no significant main effect of gender, with males and females indicating similar change in heart rate scores. There was no significant main effect of the therapy dog condition with the Therapy Dog Intervention Group and Control Group indicating similar change in heart rate scores. In addition, no significant interaction was found between gender and the intervention (See Tables 18 & 19).

**Table 18:** Descriptive statistics for heart rate.

Factor	Category	Mean	SD	N
Gender	Male	1.28	10.21	116
	Female	-0.74	9.51	82
Group	Control	-0.40	8.02	101
	Dog Intervention	0.48	11.68	97

**Table 19:** ANOVA summary table for heart rate.

Source	*df*	MS	*F*	*p*	Effect Size
A Gender	1	201.98	2.03	0.16	.01
B Group	1	1.31	0.01	0.91	< .001
A x B Interaction	1	43.15	0.43	0.51	0.002
Within groups	194				
Total	197				

*Note*.—MS  =  Mean squares, effect size  =  partial *η2*.

There were no adverse or unintended effects from participating in the therapy dog intervention.

## Discussion

Visiting therapy dog teams have been increasingly common in North American health care settings over the past decade, including for inpatient hospital stays [34, 45–47], paediatric oncology [21, 48, 49] and geriatric psychiatry [50–52]. However, there is limited research available to guide implementation generally and even less in the ED setting. Much of the research does not account for the handler, although the therapy dog and handler visit as a team. Available research has also been criticized for small sample sizes, lack of control groups and high proportions of female participants [30, 31].

With a considerable sample size, a control group, and representation of female and male participants, the current study addressed these issues and found that visiting therapy dog teams had a positive, though small, impact on patient pain and related measures of anxiety, depression and well-being. The therapy dog intervention group had a greater reduction in reported pain compared to the control group. Gender did not have a significant impact, except for a minor influence on anxiety. Heart rate and blood pressure were not impacted by the visiting therapy dog teams. The clinical significance of these findings are meaningful and require further attention and study.

### Pain

Pain is a key reason patients attend a hospital emergency department [53]. The findings in this study suggest there were significant pre-post-intervention differences for the therapy dog intervention group compared to the control. While there are no ED specific therapy dog studies with which to compare the current study findings, a beneficial impact for patients was found that was similar to studies which examined the impact of visiting therapy dogs on patient pain in a general hospital setting. In a sample of adult patients recovering from total joint replacement surgery, Havey and colleagues [54] found that according to patient medical records, those who received hospital visits from therapy dogs used significantly less pain medication than those who did not receive a visit.

Interactions with a therapy dog may alleviate pain perception by serving as a distraction from symptoms as well as influence perceptions of pain intensity [18, 34, 55]. A study by Harper et al. [23] found that involving visiting therapy dogs in client care plans immediately following joint replacement surgery improved participants’ pain scores because it assisted with distraction from the pain. Sobo and colleagues [35] propose that by providing a sufficient distraction, the interactions between a patient and a dog may not necessarily address the patient’s source of pain, but instead alleviate the perception of pain. Marcus and colleagues [56] propose a more direct relationship, suggesting that this occurs by the interaction exerting an effect on certain biological markers that correspond to pain, such as cortisol, as well as the cardiac indicators of stress, such as blood pressure and heart rate. It has also been suggested that pain reduction may be influenced by the release of beneficial hormones and neurochemicals (e.g., oxytocin), as well as decreased levels of stress hormones (i.e., cortisol) when petting an animal [57–60]. Central nervous system mechanisms may be involved through activation of endogenous pain inhibitory processes, and release of pain relieving neurochemicals such as endogenous opioids and oxytocin [59, 61, 62].

The literature on companion animals provides some context to describe the role of pets as a distraction from pain and a means of anxiety reduction in humans. Among a sample of chronic pain patients, Brown and colleagues [63] found that dog ownership provided comfort for their symptoms of pain and anxiety, which facilitated improved sleeping patterns as they were considered a constant companion and positive distraction. Similarly, Carr and colleagues [64] found that the distraction chronic pain patients’ dogs provided enabled them to self-manage their symptoms by bringing them joy and encouraging physical exercise and community engagement. Given the challenges around the management of pain in the ED are well documented and ongoing, [65] the opportunity for patients to participate in a non-pharmacological intervention, such as a therapy dog team intervention, could be very timely.

### Anxiety

This study’s findings suggest that the therapy dog intervention has a positive effect on reducing patient anxiety, and that this effect is similar for people of both male and female genders with a somewhat greater reduction in anxiety among males. It is well-established that patient anxiety is linked to the experience of pain [66, 67]. There is research suggesting that a reduction in anxiety can lead to a reduction in patient pain and increase the speed of healing [15, 68]. Eds are high anxiety settings [69]. Coakley and Mahoney [34] and others [17, 70, 71] specifically reference how a visiting therapy dog can assist patients with reducing their anxiety by inducing feelings of calm and relaxation. Hoffman and colleagues [16] identified a reduction in anxiety in patients in a hospital setting who visited with a therapy dog in their study. Other studies have relayed similar results regarding the anxiolytic effects of a visiting therapy dog in hospital settings [23]. Similar research outcomes are identified with therapy dogs in other settings, including university settings [72, 73]. Dell et al. [72] specifically found that there were minimal differences by gender, but that proportionally more females than males attended Therapy Dog visiting events to distress.

Related research also indicates that visiting with a therapy dog reduced patient anxiety and improved self-reported ratings on their experience of care at the hospital, including their interactions with hospital staff [23]. Similarly, ED staff at the Sidney & Lois Eskenazi Hospital in Indiana, USA indicated that therapy dog visits provided a cognitive distraction from work-related stressors [26].

These findings are not unique to hospital settings. Chandler [74] has incorporated therapy dogs into their mental health counselling sessions, citing several clinical gains. They note that the presence of the therapy dog contributes to a more welcoming atmosphere, and moreover, petting the dog reduces clients’ anxiety, enabling them to be more present and less guarded [73]. These observations have also attracted the employment of therapy dogs in law and justice settings, as court case witnesses may request a dog to help calm them and feel more comfortable when giving testimony [75]. Comparable outcomes have also been observed within educational settings. Among a sample of post-secondary students, Binfet [76] found that self-reported anxiety scores were significantly lower among those who interacted with a therapy dog for 20 minutes, in comparison to those in the control group. Members on our team cited similar findings when piloting a therapy dog program across multiple Canadian campuses [72].

Although the specific mechanisms underlying how interactions with therapy dogs, and more specifically therapy dog teams, affect human emotions like anxiety have yet to be identified [77], these findings are relevant to the broader literature on the human-animal bond [78]. Similar to interacting with therapy dogs, living with a pet has several benefits to alleviating a range of aversive symptoms among individuals with mental health conditions, including assistance with managing anxiety and panic attacks in both children and adults [79–81]. Through their unique way of what appears to be intuitively responding to their owners, companion animals are reported to enhance emotional states, provide emotional support, and address feelings of worry [77, 79]. The COVID-19 pandemic has brought increased attention to the support companion animals can provide to humans [82–84].

### Depression

The findings of this study suggest that the therapy dog intervention has a positive effect on reducing patient depression symptoms, and that this effect is similar for people of both male and female genders. This study’s findings align with research findings about the impact of therapy dogs visits with various populations [85], including older adults in assisted living facilities [13, 86–89] and hospitalized antepartum women with high-risk pregnancies [90]. Recent research about the relationship between pet ownership and depressive symptoms is mixed and inconclusive [64, 91–97], suggesting that the relationship between depressive symptoms and pet ownership may involve multiple factors [91]. Tower and Nokota’s [98] analysis found that among respondents to a United States internet-based survey, depressive symptoms were lowest among unmarried women living with a pet and highest among unmarried men living with a pet. More recently, others have reported the mental health benefits of living with a dog, including improved mood [99]. In relation to the COVID-19 pandemic, the growing evidence base on the impact of companion animals, including dogs, for alleviating depressive symptoms highlights several beneficial reasons for pet adoption, including helping humans cope with undesirable situations [100] and decreasing feelings of loneliness among those who live alone [101].

### Well-being

This study’s findings suggest that the therapy dog intervention has a positive effect on improving patient well-being, and that this effect is similar for people of both male and female genders. Research about humans’ experiences with therapy dog visiting and companion animals has used well-being as a general term and operationalized it in various ways: to describe physical as well as mental health, including self-reported depression and anxiety symptoms, as well as quality of life [64, 102–104]. The research is emerging on therapy dog visiting, with more available on companion animals, that includes participants’ self-reports of their overall well-being, which highlights the importance of this study’s finding that patients who visited with a therapy dog self reported an increase in well-being after their visit. These findings further illustrate the need to consider expanding therapy dog visiting, and align with the results from a recent population-based study, in which youth who did have a pet dog reported a higher rating of World Health Organization WHO-5 Well-being Index in comparison to youth who did not have a pet dog [105].

### Blood pressure & heart rate

The findings of this study suggest that the therapy dog intervention has no effect on reducing patient mean arterial blood pressure at post-30 minutes intervention, and that this effect is similar for people of both male and female genders. Similarly, the findings suggest that the therapy dog intervention has no effect on reducing patient heart rate at 30 minutes post intervention, and that this effect is also similar for people of both male and female genders. Blood pressure and heart rate are highly variable and are influenced by a wide range of variables (e.g., use of caffeine). They do not necessarily indicate a poor outcome. For example, if the therapy dog intervention participant was excited to have the therapy dog present, blood pressure and heart rate may have increased. That said, some therapy dog specific studies have found a change in both [20, 106, 107].

## Limitations & implications for future research

There are key limitations of this study that could be addressed in future research. First, the study did not specifically account separately for the impact of the handlers and therapy dogs in the visits. We did attempt, however, to standardize the handlers’ and therapy dogs’ interactions across patients as best we could. Future research in this field would be strengthened by the addition of an attention control. For example, the control group could have a handler visit for 10 minutes without the therapy dog and facilitate similar discussions about pets and animals. This would establish whether or not the animal is necessary to the success of the interaction. Second, power analysis and a larger sample would be needed to examine interaction of multiple key demographic independent variables, such as dog experience and intersecting identity factors (ethnicity and age). Third, a future study could ask control group participants during the disclosure period if they would have had been willing to visit with a therapy dog team. Based on responses, control group participants could then be excluded from the analysis who would have not met with a therapy dog team to ensure that the groups are equivalent in that regard as well. Fourth, the study was limited to one hospital setting and generalization could be strengthened with multiple hospital settings and perhaps inclusion of other provincial/territorial jurisdictions. Fifth, this study did not ask about medications taken at home that may have influenced pain scores. Information was also not collected on history of scheduled pain medication use (e.g. long-term opioid therapy) that may have influenced the responsiveness of pain to the therapy dog intervention [108]. Information was also not collected on participants who had longstanding pain (chronic pain) that may be more reticent to change with the therapy dog intervention [109]. Sixth, several factors other than the therapy dog visit could influence blood pressure and heart rate, including pacemakers or medications taken for cardiovascular disease. No data was collected on these potential influences. And seventh, given the required time commitment and other constraints of doing this study in a hectic ED, it is possible that the changes identified are not solely related to visiting with a therapy dog team and future studies should improve upon our research design. As an example, a randomized controlled design where the control condition procedures explicitly included a matched pairs design with participant pain ratings above would be an improvement.

There are several potential research and clinical implications identified from the findings of this study. With adequate access to pharmaceutical pain management a concern for ED patients, as well as long wait times, it will be important to explore creative, non-pharmaceutical options. There is also heightened concern with pain medication misuse, and specifically in light of Canada’s public health opioid crisis [110, 111]. Patient waiting has also been associated with negative emotional states and well-being in ED patients [5]. Negative feelings, particularly anxiety and stress, can be intensified when patients encounter uncertainty regarding their pain [112]. The role of therapy dog visits in decreasing patients’ perceived pain, whether as a distraction or by some other mode, is an important finding that should be examined further in both practice and research. Related, an area for further examination is central nervous system mechanisms that can assist in explaining the reduction in participant pain, depression and anxiety and improvement in well-being. This can include the role of memories, especially when engaging patients in conversation about their pets. For example, members of our team undertook a study of the effect of therapy dogs on the wellbeing of older Veterans living in a long-term care residence. We found that the therapy dog visits had “a positive influence on memory recollection and reminiscence among [V]eterans” (p. 83) [113].

## Conclusion

Decreasing patient pain is an important health issue for Canadian EDs. This research is one of a handful of ED specific visiting therapy dog studies, and the only one known to these authors to focus on therapy dog team visits, patient pain, anxiety, depression, and well-being. It is also one of a limited number of controlled study designed studies in the animal assisted intervention field. It follows that observing a clinically significant change in pain, as well as significant changes in anxiety, depression and well-being in the therapy dog intervention compared to the control group in this study is an important contribution to the literature and for future research and practice.

## Supporting information

S1 Checklist CONSORT 2010 checklist. (PDF)

S1 File Behavioral application. (DOCX)
