# Supplementary material for: Outcomes of a controlled trial with visiting therapy dog teams on pain in adults in an emergency department
Source: PLoS One. 2022 Mar 9;17(3):e0262599. doi: 10.1371/journal.pone.0262599 (PMC9064456; doi:10.1371/journal.pone.0262599)
Supplement: S1 File — (DOCX) [file pone.0262599.s002.docx]

| **Behavioural Application** | **For Internal Use Only** |
| --- | --- |
|  | **UnivRS Internal ID:**  **Date Received:** Click here to enter a date. |

**Part 1: Key Information**

| Title*: A Study of the ‘Pawsitive’ Impacts of Therapy Dog Visits with Adult Emergency Department Pain Patients  Level of Risk: * Minimal risk  Expected Start Date: * 2019-06-01  Expected End Date: * 2019-11-30  If applicable, explain why this application is time sensitive: **The RUH ED will move to its new location in November, 2019. We cannot collect data in two different locations with our control design. Also, our SCPOR funded researcher organizing the study and the data collection lead is only funded until November, 2019. We just received notice of our funding at the start of this month.** |
| --- |

**Project Personnel**

| **Principal Investigator**   \| **Name:** \| **NSID:** \| **Email:** \| **Phone:** \| **Organization (Department):** \| \| --- \| --- \| --- \| --- \| --- \| \| **Colleen Anne Dell** \| **Cad924** \| [Colleen.dell@usask.ca](mailto:Colleen.dell@usask.ca) \| **5912** \| Sociology \|   **Sub-Investigator(s)**   \| **Name:** \| **NSID:** \| **Email:** \| **Phone:** \| **Organization (Department):** \| \| --- \| --- \| --- \| --- \| --- \| \| James Stempien \| **Jas510** \| Stempien@islandnet.com \| 306-220-8618 \| Emergency Medicine \| \| Eloise Carr \| **N/A** \| ecarr@ucalgary.ca \| 403-220-6267 \| Faculty of Nursing \| \| Susan Tupper \| **Smt677** \| Susan.tupper@usask.ca \| 306-655-1041 \| Saskatchewan Health Authority \| \| Peter Butt \| **Prb984** \| Peter.butt@usask.ca \| 306-655-0452 \| U of S Medicine  (Family medicine) \| \| Donna Goodridge \| dpg031 \| Donna.goodridge@usask.ca \| 306-966-4209 \| U of S Medicine \| \| Jane Smith \| **N/A** \| smithpj@sasktel.net \| 306-491-4857 \| St. Johns Ambulance, Royal University Hospital \| \| Lindsey Broberg \| **Lja766** \| Lja766@mail.usask.ca \| 306-261-7396 \| Obstetrics and Gynecology Resident \| \| Benjamin Carey \| **Bdc114** \| Bdc114@mail.usask.ca \| 306-380-7338 \| Sociology \| \| Alicia Husband \| **Akh671** \| alicia.husband@usask.ca \| 966-6717 \| Sociology \| \| Lacey Jerk \| **Lcj050** \| lcj050@mail.usask.ca \|  \| College of Medicine \| \| Beverly Morrison \| **Bjm194** \| Bjm194@mail.usask.ca \| 966-7237 \| Veterinary Microbiology \| \| Betty Rohr \| **Bar123** \| Betty.rohr@usask.ca \| 966-8548 \| Sociology \|   **Student(s)**   \| **Name:** \| **NSID:** \| **Email:** \| **Phone:** \| **Organization (Department):** \| \| --- \| --- \| --- \| --- \| --- \| \|  \|  \|  \|  \|  \|   **Primary Contact**   \| **Name:** \| **NSID:** \| **Email:** \| **Phone:** \| **Organization (Department):** \| \| --- \| --- \| --- \| --- \| --- \| \|  \|  \|  \|  \|  \|   **Secondary Contact**   \| **Name:** \| **NSID:** \| **Email:** \| **Phone:** \| **Organization (Department):** \| \| --- \| --- \| --- \| --- \| --- \| \|  \|  \|  \|  \|  \| |
| --- | --- | --- | --- | --- | --- | --- | --- | --- | --- | --- | --- | --- | --- | --- | --- | --- | --- | --- | --- | --- | --- | --- | --- | --- | --- | --- | --- | --- | --- | --- | --- | --- | --- | --- | --- | --- | --- | --- | --- | --- | --- | --- | --- | --- | --- | --- | --- | --- | --- | --- | --- | --- | --- | --- | --- | --- | --- | --- | --- | --- | --- | --- | --- | --- | --- | --- | --- | --- | --- | --- | --- | --- | --- | --- | --- | --- | --- | --- | --- | --- | --- | --- | --- | --- | --- | --- | --- | --- | --- | --- | --- | --- | --- | --- | --- | --- | --- | --- | --- | --- | --- | --- | --- | --- | --- |

**Sponsor(s)**

| \| **Sponsor:** \| **Pending / Awarded** \| \| --- \| --- \| \| **RUH Foundation** \| **20,000** \| |
| --- | --- | --- | --- | --- |

**Agency(ies)**

| \| This project is funded: * \| Yes  No \| \| --- \| --- \| \| The funding supporting this project will be administrated at the University of Saskatchewan: \| Yes, complete Part A  No, complete Part B \|   **Part A: For Grants and Contracts administered by the U of S:**  Project Application(s) Directly Associated with the Fund(s) Supporting this Project  Specify the UnivRS internal ID# (for pending grants or contracts):  Project(s) Directly Associated with the Fund(s) Supporting this Project  Specify the UnivRS internal ID# (for awarded grants or contracts):  **Part B: For Grants or Contracts not administered by the U of S:**   \| **Agency:** \| **Pending / Awarded** \| \| --- \| --- \| \| **RUH Foundation** \| **20,000** \| |
| --- | --- | --- | --- | --- | --- | --- | --- | --- |

**Location(s) Where Research Activities Are Conducted**

| Enter every location where this research will be conducted under this Research Ethics Approval: * Royal University Hospital Emergency Department  Country(ies):* List all countries where you will be conducting your research under this Research Ethics Approval: Canada  If this project will be conducted within schools, health regions, or other organizations, specify how you will obtain permission to access the site. Submit a copy of the certificate or letter of approval when obtained. Letter of approval has been obtained (see attached)  If you do not plan to seek approval, provide a justification: |
| --- |

**Other Ethics Approval**

| \| This project has applied for/received approval from another Research Ethics Board(s) * \| Yes  No \| \| --- \| --- \|   If 'yes', identify the other Research Ethics Board(s): |
| --- | --- | --- |

**Conflict of Interest**

| Confirm whether any member of the research team or their immediate family members will:   \| Receive personal benefits over and above the direct costs of conducting the project, such as remuneration or employment: * \| Yes  No \| \| --- \| --- \| \| Receive significant payments from the Sponsor such as compensation in the form of equipment, supplies or retainers for ongoing consultation and honoraria: * \| Yes  No \| \| Have a non-financial relationship with the Sponsor such as unpaid consultant, board membership, advisor or other non-financial interest: * \| Yes  No \| \| Have any direct involvement with the Sponsor such as stock ownership, stock options or board membership: * \| Yes  No \| \| Hold patents, trademarks, copyrights, licensing agreements or intellectual property rights linked in any way to this project or the Sponsor: * \| Yes  No \| \| Have any other relationship, financial or non-financial, that if not disclosed, could be construed as a conflict of interest: * \| Yes  No \|   If yes was answered to any question(s), explain the personal benefit(s) and how the conflict will be managed: |
| --- | --- | --- | --- | --- | --- | --- | --- | --- | --- | --- | --- | --- |

**Part 2: Project Overview**

**Project Overview**

| *Summarize this project, its objectives and potential significance:* The goal of this unique 18 month study is to better understand the experiences of pain patients in the Royal University Hospital (RUH) Emergency Department (ED) to create excellence in health care. The purpose is to measure the impact of visiting therapy dogs on reducing ED patient pain. The background rationale is that pain is the primary reason individuals attend an ED, patient pain is generally not well managed in EDs, Saskatchewan EDs have among the longest wait times in the country, and anxiety associated with ED waiting can negatively impact patients’ pain. Research suggests a therapy dog can change patients’ perceptions of pain and its intensity and facilitate relaxation. The intervention will be examined for its impact on patients’ sensory pain (i.e., physical pain severity), affective pain (i.e., emotional pain unpleasantness) and anxiety. It is important to find creative, low-cost ways to respond to patients attending the ED for pain. The primary objective of this study is to generate new health-related knowledge on the ED pain patient experience. The secondary objectives are to implement effective end-of-grant knowledge translation and dissemination strategies and undertake a successful model of collaborative, multidisciplinary research among researchers, patient advisors and system representatives, rooted in a One Health framework.  *Provide a description of the research design and methods to be used:*  St. John Ambulance Therapy Dog teams are comprised of a certified (tested and passed) therapy dog and handler. The goal of the St. Johns Ambulance therapy dog program is to offer support and comfort to individuals the dogs visit with. Five therapy dog teams commonly visit the RUH ED five days a week. The visits follow a standardized protocol (i.e., sign in, patients interact with the therapy dog, handlers share information about the dog). In this study therapy dogs will visit a convenience sample of consenting RUH ED pain patients five days a week. Each therapy dog team will participate once-a-week, for a 90 minute period, and during peak and low patient ED wait times (by day of the week and time). Participants will be over the age of 18, able to provide consent, attending the ED because of pain and want to visit with a therapy dog. These patients are located in the ‘active area’ of the ED in individual curtained-off spaces with beds; the patients are either waiting to be seen by a physician, have treatment in progress, or are admitted to hospital and waiting for a unit bed. A control group will also be involved, with the same process and sample.  Data will be collected 3 days/ week (alternating with control days). Data will be collected in five steps, using quantitative psychometric measures that will not exceed 10 minutes. Patient pain will be measured with the Edmonton Symptom Assessment System (revised version) (ESAS-R) (numerical pain scale) and patient anxiety will be measured with biomarkers (heart rate, blood pressure) and the ESAS-R (anxiety, depression and overall wellbeing questions). The Human-Animal Interaction Scale (HAIS) will be applied to document the behaviour and amount of interaction between the therapy dog and patient. Quantitative data analysis using both descriptive and inferential statistical procedures will be conducted to examine distributions, relationships and differences among variables. Measures of outcome variables will be compared between pre- and post-therapy dog intervention and the influence of moderator variables, such as gender, will be examined. The HAIS score will indicate the quantity of positive human-animal interaction.  Control group data will be collected 2 days/ week (alternating with animal testing days) with a goal of 8 samples per day. The data collection will occur in two stages. First, patient pain will be measured with the Edmonton Symptom Assessment System (revised version) (ESAS-R) (numerical pain scale) and patient anxiety will be measured with biomarkers (heart rate, blood pressure) and the ESAS-R (anxiety, depression and overall wellbeing questions). The HAIS scale will not be administered during the control as there will be no animal interaction. Control days require two research assistants who will obtain consent using a deceptive consent form which will explain that this is a pain study but will omit any information regarding therapy dogs. Once all measures are taken the participants will be explained the deception and asked to sign the true consent form.  All of our team members will meet on a monthly basis. The team represents a solid compliment of expertise, experience and knowledge (contributing from data collection through to end-of-grant knowledge translation). This will enable the team to fully deliver on the project goal, purpose and objectives. The outcomes of this study will be meaningfully translated and disseminated to improve the health care of RUH ED pain patients with peer reviewed publishing, RUH monitor adverts and webinar, and a news release. The clinical relevance of this study is that the outcomes will offer a complementary understanding of quality health care for ED pain patients. To date there have been no studies of therapy dogs in EDs in Canada. |
| --- |

**Duration and Location of Data Collection Events**

| Outline the duration and location of data collection for the following, if applicable:  Audio/Video Recording(s):  Ethnography:  Focus Group(s):  Group Interview(s):  Home Visit(s):  Individual Interview(s):  *Non-Invasive Physical Measurement(s):* Anxiety will be measured using biomarkers: heart rate and blood pressure. All measurements will be taken by a trained RA before the visit, during and 15 minutes after the visit.  Participant Observation:  *Questionnaire(s):* Once a candidate is identified and before the visit takes place, participants will be  asked by the RA to complete the pain, anxiety and demographic questions (Edmonton Symptom Assessment System (revised version) (ESAS-R) (numerical pain scale) (see attached). After the therapy dog visit the patient will be asked to complete the HAIS. Fifteen minutes post visit the participant will be asked to complete the pain and anxiety questions for a second time.  Secondary Use of Data or Analysis of Existing Data:  Other: |
| --- |

**Internet-Based Interaction**

| \| Confirm whether this project will involve internet-based interactions with participants, including e-mails: * \| Yes  No \| \| --- \| --- \|   If a third party research or transaction log tool, screen capturing or website survey software or masked survey site is used, describe how the security of data gathered at those sites will be ensured:  Describe how permission to use any third party owned site(s) will be obtained:  If participants may be identified by their email address, IP address or other identifying information, explain how this information will remain private and confidential: |
| --- | --- | --- |

**Anonymity and Confidentiality**

| \| Confirm whether participants will be anonymous in the data gathering phase of the project: * \| Yes  No \| \| --- \| --- \|   If 'No' was answered to the previous question, explain how the confidentiality of participants and their data will be protected, and include whether the research procedures or collected information may reasonably be expected to identify an individual:  All participants will be given an identifier on their data collection sheets, and no identifying information will be collected.  Identify any factors that may limit the researchers’ ability to guarantee confidentiality:   \| Limits due to the nature of group activities, such as a focus group where the project team cannot guarantee confidentiality: \| Yes  No \| \| --- \| --- \| \| Limits due to context: individual participants could be identified because of the nature or size of the sample: \| Yes  No \| \| Limits due to context: individual participants could be identified because of their relationship with the project team: \| Yes  No \| \| Limits due to selection: procedures for recruiting or selecting participants may compromise the confidentiality of participants, such as those referred to the project by a person outside the project team: \| Yes  No \|   Other confidentiality limits: |
| --- | --- | --- | --- | --- | --- | --- | --- | --- | --- | --- |

**Risks and Benefits**

| Explain the psychological, emotional, physical, social or legal harms that participants may experience during or after their participation: The study has three minor ethical issues. First is the perceived risk of zoonotic disease transmission in the ED.  Second is the potential negative patient reaction to a therapy dog presence in the ED.  Third, there may be some minimal discomfort experienced by patients in taking their anxiety biomarkers.  Describe how the above risks will be managed. If appropriate, identify any resources to which they can be referred:  First, during the pilot study the team applied the American Veterinary Medical Association therapy dog visiting guidelines and we developed RUH ED specific visiting protocols. These will apply to the proposed study. We also have the expertise of Dr. Joe Rubin (Professor Veterinary Microbiology) on our research advisory group.  Second, Policies and procedures have been developed to ensure the health and safety of patients, staff, therapy dogs and handlers (e.g., approaching patients upon consent, signage in the ED). Our team also conducted a patient study in the ED waiting room in 2017 and found that more than 80% of participants agreed or strongly agreed that people would want to visit a therapy dog.  Third, the discomfort experienced by patients in taking their anxiety biomarkers is minimal and not out of the ordinary of regular ED procedures.  IT IS IMPORTANT TO NOTE that the therapy dogs regularly visit the RUHD ED, and have been doing so for three years, irrespective of this study.  Describe the likely benefits of the research that may justify the above risk(s): The clinical relevance and benefits of this study to RUH pain patients is grounded in recognition of the potential benefits of animals in human pain patient health care in the ED. Examining the impact of a unique psychosocial health intervention on pain patients’ experiences in a hospital ED can offer a complementary understanding of quality health care. There have been no studies of therapy dogs in EDs in Canada, one opinion survey in the US has been undertaken, and a recent (2019) controlled trial in a US ED department with therapy dogs. The US opinion study found that “93% of patients and 95% of staff agreed that therapy dogs should visit EDs". This finding highlights what is widely accepted in the companion animal literature but infrequently by ED medicine and medicine generally; interacting with animals can benefit human bio-psycho-social-spiritual health. The controlled study found that “Exposure to therapy dogs plus handlers significantly reduced anxiety in ED patients”. |
| --- |

**Part 3: Community Engagement**

**Aboriginal Peoples and Community Engagement**

| \| Aboriginal communities, peoples, language, culture or history is the primary focus of this project: * \| Yes  No \| \| --- \| --- \| \| Aboriginal people will comprise a sizable proportion of the larger community that is the subject of research even if no Aboriginal-specific conclusions will be made: * \| Yes  No  Not Applicable \| \| There is an intention to draw Aboriginal-specific conclusions from this project: * \| Yes  No \| \| This project will involve community-based participatory research: * \| Yes  No \| \| There will be a research agreement between the researcher and community: \| Yes  No \| |
| --- | --- | --- | --- | --- | --- | --- | --- | --- | --- | --- |

**Aboriginal Engagement and Community-Based Participatory Research**

| If 'yes' was answered to any of the above questions, complete the following:  Outline the process to be followed for consulting with the appropriate community:  Describe the organizational structure and community processes required to obtain approval within the specific community(ies):  Describe any customs and codes of research practice that apply to the particular community(ies) affected by the project:  Describe how the research plan will consider mutual benefit to the participating community(ies), support capacity building through enhancement of the skills of community personnel and the recognition of the role of elders and other knowledge holders:  Describe how the community representatives will have the opportunity to participate in the interpretation of the data and the review of research findings before the completion of any reports or publications:  Describe how the final project results will be shared with the participating community(ies): |
| --- |

**Part 4: Recruitment and Consent**

**Participant Recruitment**

| *Indicate the expected number of participants and provide a brief rationale for the number:* A sample size of at least 122 in both the control and animal intervention is required to detect statistically significant results based on information collected on patient response in the pilot study.  *Describe the criteria for including participants:*  Participants will be over the age of 18, able to provide consent, attending the ED because of pain and want to visit with a therapy dog. These patients are located in the ‘active area’ of the ED in individual curtained-off spaces with beds; the patients are either waiting to be seen by a physician, have treatment in progress, or are admitted to hospital and waiting for a unit bed. Participants will also be willing to visit with a therapy dog team (although the control group will not be visiting with a dog).  *Describe the criteria for excluding participants:*  The RA will access the patient’s Medication Administration Record (MAR) to confirm non-administration of pain medications (specifically immediate release acetaminophen and opioid analgesics) within the hour prior to the visit. This step is included to prevent confounding the potential effect of the therapy dog intervention on pain outcomes.  *Provide a detailed description of the method of recruitment, such as how and whom will identify and contact prospective participants:* RA #1 will identify patients who are verbally coherent and interested in visiting with a therapy dog. Consent will be obtained from pain patients to participate in the study. All ED patients can visit with a therapy dog, regardless of study participation. The RA will access the patient’s Medication Administration Record (MAR) to confirm non-administration of pain medications (specifically immediate release acetaminophen and opioid analgesics) within the hour prior to the visit. This step is included to prevent confounding the potential effect of the therapy dog intervention on pain  *Outcomes.*  If the project involves vulnerable, distinct, or cultural groups, or if the project is above minimal risk, describe the research team's experience or training in working with the population: **N/A**  *Explain any relationship between the researchers and the participants, including any safeguards to prevent possible undue influence, coercion or inducement:* All questions are pre written and many are established instruments such as the Human Animal Interaction questionnaire. The participant can also have the option of filling out the questions on their own. Participant answers and identity will be kept anonymous.  *Provide the details of any compensation or reimbursements offered to the participants:* **N/A** |
| --- |

**Consent Process**

| *Describe the consent process:*  Consent form attached, RA #1 will explain the form and how the information gathered will be used.  *Specify who will explain the consent form and consent participants:* Research assistant #1 will explain the form before the therapy dog visit is conducted. If the individual does not consent to the research they will still be allowed to visit with the therapy dog.  *Explain where and under what circumstances consent will be obtained from participants:*  Consent will be obtained from each person before the data is collected, and before the therapy dog visit.  *Describe any situation where the renewal of consent might be appropriate and how it may be*  *obtained:* * **N/A**  *If deception of any kind will be used, justify its use, describe the protocol for debriefing and re-consenting participants upon completion:* Deception will be used on days where control data is collected. Participants will sign a consent form that indicates the study they are signing off on will be about the impacts of psychosocial intervention in emergency pain patients. The deception is used to guarantee the patients pain and anxiety be unaffected by any knowledge of therapy dogs or that they are part of a control group. Once all the data needed is gathered from the participant they will be explained the deception and given another consent form to sign with the true information regarding the study.  *If any of the participants are not competent to consent, describe the process by which their capacity or competency will be assessed, identify who will consent on his/her behalf (including any permission or information letter to be provided to the person or persons providing alternate consent), as well as the assent process for participants:* **N/A**  *Describe how and when participants will be informed about their right to withdraw, including the procedures to be followed for participants who wish to withdraw at any point during the project*: Participants will be informed about their right to withdrawal at the time the consent form is given and explained. Participants can withdraw at any point from the study up until the data is analyzed. |
| --- |

**Part 5: Security and Storage**

**Data Security and Storage**

| Identify the research personnel responsible for data collection:  Benjamin Carey and Alicia Husband  Specify who will have access to raw data, which may include information that would identify participants:  Colleen Dell, Benjamin Carey and Alicia Husband  Describe the data storage plans, including the arrangements for preventing the loss of data:  Data will be uploaded to an external hard drive and all paper data will be stored in a locked container.   \| Confirm whether the Principal Investigator will be responsible for data storage: * \| Yes  No \| \| --- \| --- \|   If no, specify the reasons and indicate who will be responsible for data storage:  Specify how long data will be retained:  5 years minimum as per University of Saskatchewan Guidelines  If other, specify duration and provide justification:  Explain how the collected data is intended to be published, presented, or reported:  Peer-reviewed article writing, presentation at the Canadian Association of Emergency Physicians conference (if external funding is secured), and end-of-grant knowledge translation.  Describe the final disposition of research materials:  *Any paper copies will be shredded and saved data will be destroyed (erased).   \| State whether data will be transferred to a third party: * \| Yes  No \| \| --- \| --- \|   Organization(s) where data will be transferred:  Indicate how data will be transferred to the third party: Choose an item.  If other, please specify: |
| --- | --- | --- | --- | --- |

**Part 6: Declaration of Principal Investigator**

| By submitting this application form, the Principal Investigator (PI) attests to the following:   - the information provided in this application is complete and correct. - the PI accepts responsibility for the ethical conduct of this project and for the protection of the rights and welfare of the human participants who are directly or indirectly involved in this project. - the PI will comply with all policies and guidelines of the University and affiliated institutions where this project will be conducted, as well as with all applicable federal and provincial laws regarding the protection of human participants in research. - the PI will ensure that project personnel are qualified, appropriately trained and will adhere to the provisions of the Research Ethics Board-approved application. - that adequate resources to protect participants (i.e., personnel, funding, time, equipment and space) are in place before implementing the research project, and that the research will stop if adequate resources become unavailable. - any changes to the project, including the proposed method, consent process or recruitment procedures, will be reported to the Research Ethics Board for consideration in advance of implementation. - will ensure that a status report will be submitted to the Research Ethics Board for consideration within one month of the current expiry date each year the project remains open, and upon project completion. - if personal health information is requested, the PI assures that it is the minimum necessary to meet the research objective and will not be reused or disclosed to any parties other than those described in the Research Ethics Board-approved application, except as required by law. - if a contract or grant related to this project is being reviewed by the University or Health Region, the PI understands a copy of the application, may be forwarded to the person responsible for the review of the contract or grant. - if the project involves Health Authority resources or facilities, a copy of the ethics application may be forwarded to the Health Authority research coordinator to facilitate operational approval. |
| --- |

**Document(s)**

Please provide a list of documents that are being submitted along with this application: e.g. Consent forms, questionnaires, interview questions, data collection sheets, recruitment materials.  **Consent form (visit with the Therapy Dog)
Consent form (not visit with the Therapy Dog)
St. John Ambulance Therapy Dog Program Letter of Support
Saskatoon Health Region Letter of Support
Data collection tools
-ESAS-R (indicate on picture; scale 1-10)
-Main reason for attending the ED
-Heart rate and blood pressure
-ESAS-R numerical scale (anxiety, depression, wellbeing)
-Patient demographics
-HAIS form**
